# Visually Driven Activation in Macaque Areas V2 and V3 without Input from the Primary Visual Cortex

**DOI:** 10.1371/journal.pone.0005527

**Published:** 2009-05-13

**Authors:** Michael C. Schmid, Theofanis Panagiotaropoulos, Mark A. Augath, Nikos K. Logothetis, Stelios M. Smirnakis

**Affiliations:** 1 Max Planck Institut für biologische Kybernetik, Tübingen, Germany; 2 Imaging Science and Biomedical Engineering, University of Manchester, Manchester, United Kingdom; 3 Departments of Neuroscience and Neurology, Baylor College of Medicine, Houston, Texas, United States of America; University of Regensburg, Germany

## Abstract

Creating focal lesions in primary visual cortex (V1) provides an opportunity to study the role of extra-geniculo-striate pathways for activating extrastriate visual cortex. Previous studies have shown that more than 95% of neurons in macaque area V2 and V3 stop firing after reversibly cooling V1 [1], [2], [3]. However, no studies on long term recovery in areas V2, V3 following permanent V1 lesions have been reported in the macaque. Here we use macaque fMRI to study area V2, V3 activity patterns from 1 to 22 months after lesioning area V1. We find that visually driven BOLD responses persist inside the V1-lesion projection zones (LPZ) of areas V2 and V3, but are reduced in strength by ∼70%, on average, compared to pre-lesion levels. Monitoring the LPZ activity over time starting one month following the V1 lesion did not reveal systematic changes in BOLD signal amplitude. Surprisingly, the retinotopic organization inside the LPZ of areas V2, V3 remained similar to that of the non-lesioned hemisphere, suggesting that LPZ activation in V2, V3 is not the result of input arising from nearby (non-lesioned) V1 cortex. Electrophysiology recordings of multi-unit activity corroborated the BOLD observations: visually driven multi-unit responses could be elicited inside the V2 LPZ, even when the visual stimulus was entirely contained within the scotoma induced by the V1 lesion. Restricting the stimulus to the intact visual hemi-field produced no significant BOLD modulation inside the V2, V3 LPZs. We conclude that the observed activity patterns are largely mediated by parallel, V1-bypassing, subcortical pathways that can activate areas V2 and V3 in the absence of V1 input. Such pathways may contribute to the behavioral phenomenon of blindsight.

## Introduction

The flow of visual information from the retina to the higher cognitive and motor areas contains a series of transformation steps involving a number of subcortical and cortical brain areas. The finding that, along this path, the receptive fields of neurons increase in size, become step by step more invariant to physical dimensions, and come to reflect, in part, perceptual processes has contributed to the view that visual information processing is largely organized in a serial hierarchical fashion. The primary visual cortex (V1) is considered to be the major entry point for cortical visual processing and activity in subsequent “higher” visual areas is generally interpreted as arising primarily from a transformation of V1 input. Although this model has been highly successful for studying how visual information is transformed across areas, it is clear that it provides only a partial description of the actual information flow. This is corroborated by anatomical studies, which have revealed the existence of numerous parallel and feedback inter-areal connections [4] whose functional significance remains largely unknown. For example, in addition to V1 input, most extra-striate areas receive direct, V1-bypassing, input from the thalamic nucleus of the Pulvinar as well as from the lateral geniculate nucleus (LGN). To date the role that these pathways play in visual processing is at best poorly understood.

The importance of parallel, V1-bypassing, pathways for mediating residual visual function following striate cortical lesions has become evident from the large number of studies on human patients and monkeys with area V1 lesions that exhibit so-called “blindsight”-behavior (see reviews [5], [6]): Despite the presence of a scotoma in the lesion affected part of the visual field, subjects of both species perform above chance on certain visual detection and discrimination tasks under forced choice conditions [7], [8], [9], [10], [11], [12], and at times are even able to report the perceptual qualities of the stimulus [13], [14]. Studying the patterns of activity that persist in extrastriate areas following area V1 lesions is a first step in trying to understand how the visual cortex adjusts to injury, and in trying to identify candidate areas that might mediate the residual visual behavior observed in the phenomenon of “blindsight”.

All cortical visual areas studied so far (V2, V3, V3A, V4, V5/MT, MST, STP, IT) are clearly dependent on V1 input, so that, upon lesioning or inactivating V1 the majority of cells in these areas either cease to respond or show a markedly reduced response to the visual stimulus [1], [2], [3], [15], [16], [17], [18], [19], [20], [21], [22], [23], [24]. Neuronal responses in areas V2, V3, depend particularly strongly on V1 input as more than 95% of recording sites in these areas lose visual modulation following transient V1 inactivation by cooling [1], [3], [25]. Areas V4 [18] and IT [21] are similar to V2,V3 in this regard, whereas V3A [3], V5/MT+ [15], [19], [22], [23] (but see [16], [17], [24]), and STP [26] retain considerable visual responsiveness following V1 lesions. Although these studies [1], [2], [3], [18] suggest that early extra-striate areas V2 and V3 get effectively silenced immediately following V1 inactivation, it remains unknown whether visual responsiveness can return in these areas over time following a *permanent* V1 lesion.

Neuroimaging studies confirmed the preservation of V5/MT+ activity in two highly studied human patients who exhibit “blindsight” [27], [28], [29] following V1+ lesions. By contrast, results describing the function of visual areas V2 and V3 after long-standing V1 lesions are limited: 1) there are no electrophysiological studies addressing directly this issue in monkeys, and 2) neuroimaging studies of human “blindsight” patients have not produced definitive results: On the one hand, the fMRI study of hemianopic patients FS and GY by Goebel et al., appears to confirm results from primate electrophysiology suggesting that visually driven activity in areas V2, V3 is strictly dependent on V1 input [29]. In contrast, Baseler et al., report that visually driven activity persists in areas V2, V3 and V3A [30] of patient GY, and argue that this is likely the result of cortical reorganization. The difference in these reports underscores how difficult it is to derive definitive conclusions by studying naturally occurring human V1 lesions, which tend to be highly variable, extending typically into the underlying white matter and into areas V2, V3 while potentially leaving “islands” of V1 cortex intact. In particular, the lesion of patient GY suffers from both these problems [30], complicating the interpretation of the findings described above.

These important isolated reports notwithstanding, no study to date has systematically followed how the strength of visual modulation in primate extrastriate cortex evolves in time from pre-lesion levels following isolated V1 lesions. Here we use functional magnetic resonance imaging (fMRI) blood oxygen level dependent (BOLD) signal measurements to serially monitor the activity patterns seen after isolated V1 lesions in the retinotopically corresponding locations of macaque areas V2 and V3 (V2, V3 lesion projection zones or LPZs). Macaque BOLD signal measurements are well suited for addressing this issue because they are non-invasive, they can provide a high spatial (∼1 mm) resolution picture of global brain activity [31], [32] and co-localize to within ∼1 mm with recorded multi-unit activity [33]. In what follows we will try to address the following questions: 1) do areas V2, V3 display visually driven modulation in the absence of V1 input (i.e. are they in principle capable of mediating aspects of “blindsight” behavior), 2) if so, what can we say about the V1-bypassing pathways that give rise to the persisting activity, and 3) does the pattern of the observed activity change in time suggesting that cortical reorganization takes place? Although currently at a premature stage, information gathered by studies of this type may in the future be helpful for designing interventions that may accelerate recovery following visual system injury.

## Results

To examine the dependence of visually driven activity in areas V2 and V3 on input from V1, we performed fMRI experiments in two anesthetized monkeys (L02 & Q02) with area V1 lesions induced by aspiration (Materials and Methods). A standard phase encoding expanding ring stimulation paradigm was used to map the retinotopic (eccentricity) organization in areas V2 and V3. Baseline experiments were performed prior to lesioning, and subsequently the animals were scanned starting at 1 month post lesioning and reaching up to 277 days (Q02) and 681 days (L02) post-lesioning.

### Characterization of the V1 lesion

**Figure 1:**
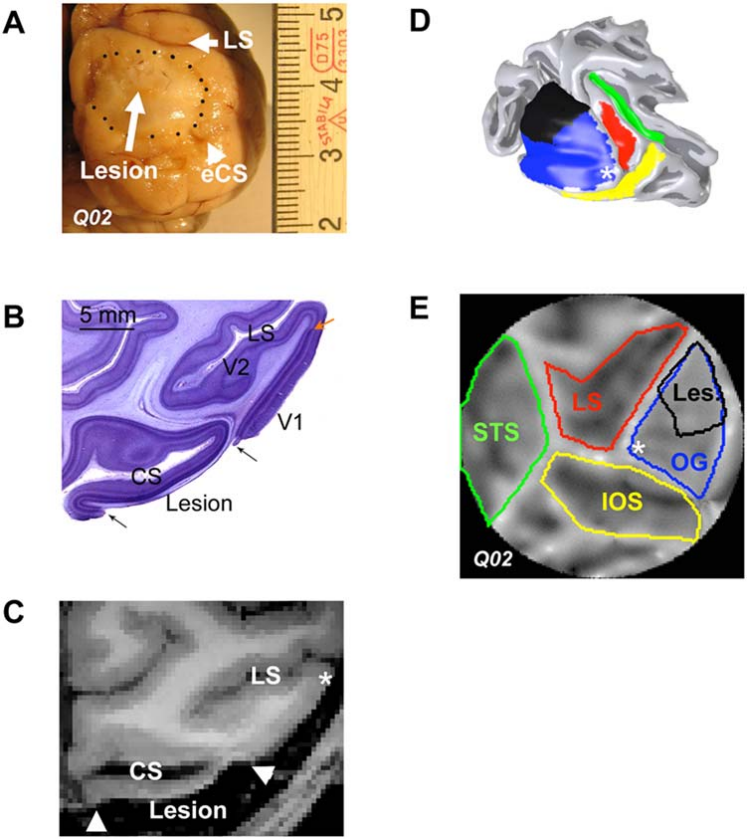
Characterization of the V1 Lesion. A Picture of Macaque Q02's brain post-mortem. The area of completely denuded gray matter and exposed white matter, is highlighted in the picture by black dots. The dorsal border of the lesion approaches the lunate sulcus (LS) and the ventral border reaches up to the external calcarine (eCS). Major ticks of the scale bar are in centimetres. B Nissl stained axial section (100 µm thick) through the center of Q02's V1 lesioned cortex. Arrows point to the borders of the V1 lesion, which completely destroyed gray matter but largely spared the underlying white matter. Note the characteristic line of Gennari indicating the border between V1 and V2 (red arrow). The V1 cortex surrounding the lesion and the lunate sulcus (LS) containing areas V2 and V3 are not affected by the lesion. C Axial MRI slice through the visual cortex of macaque Q02. The lesioned area is evident in the MR image due to the absence of gray matter. D 3-D reconstruction of the surface of the visual cortex of macaque Q02. The 3D rendering represents the border between gray and white matter. Some of the prominent anatomical landmarks are color coded for easier visualization in the flat map view (see panel E). The V1 lesion shown in black was reconstructed by manually selecting the area devoid of gray matter (panel C). Dorso-ventrally, it starts 1–2 mm ventral to the lunate reaching up to the external calcarine sulcus, and medio-laterally from the edge of the internal calcarine sulcus to ∼14 mm from the intersection of the lunate and inferior occipital sulci (*, fovea). E Flat map of the visual cortex of Macaque Q02. Sulci and gyri are shown as dark and light regions respectively. Sulci and gyri of visual cortex are color-coded in the same way as in the 3-D reconstruction of panel D. Abbreviations: LS: lunate sulcus, eCS: external calcarine sulcus, CS: internal calcarine sulcus, IOS: inferior occipital sulcus, OG: occipital gyrus, STS: superior temporal sulcus Les: Lesion.

In both monkeys, lesions were located in the right dorsal V1 sandwiched between the lunate (LS) and the external calcarine (eCS) sulci (Figure 1A). Care was taken to aspirate gray matter completely while leaving white matter as unaffected as possible. Histological analysis in one monkey (Q02) confirmed that the lesion was complete with no residual gray matter found inside the border of the lesion (Figure 1B), while white matter remained largely intact. Note that damage in the white matter could potentially undercut additional input pathways to V2 (or V3) and could potentially cause the loss of BOLD signal there. However, complete loss of the BOLD signal is not what we observed. Gray matter immediately (within 1 mm) surrounding the V1 lesion was histologically (Q02) and radiologically (Q02 and L02) intact and showed reliable visually driven BOLD responses (see below). The loss of V1 gray matter was clearly detectable in the MR anatomical images (0.5 mm^3^ resolution anatomical Mdeft scan [34], [35]; Figure 1C), so that the lesioned area could be selected independently of functional activity as an ROI for further analysis. The cortical gray matter ribbon lining the lunate sulcus, including the lesion projection zone of areas V2 and V3, was intact. To measure the extent of the lesion and the lesion projection zone in pre-striate areas, we reconstructed 3D renderings of the surface of the visual cortex (Figure 1D) and converted them into cortical flat maps (Figure 1E). The extent of the lesion was similar in both monkeys: Along the lateral-to-medial axis the lesion started at 16 mm (L02) or 14 mm (Q02) from the foveal representation extending laterally by 16 mm (L02) or 13 mm (Q02), up to the lip of the calcarine sulcus (area V1 of Q02 was smaller than L02). Along the dorsal-to-ventral axis the lesions were situated between the lunate and the external calcarine sulcus covering a distance of ∼17 mm in both monkeys. This resulted in a lower quadrant visual field scotoma extending from ∼2–7° for both monkeys L02 and Q02. The distance of the lesion from the lunate sulcus was ∼1–2 mm and may therefore have affected a small portion of the opercular part of area V2. Independent experiments using a meridian mapping paradigm as in [36] confirmed that the lesion included the horizontal meridian which ensures that the entire dorsal portion of V1 was lesioned at the specified eccentricities. The V1 area covered by the lesions was 235 mm^2^ for L02 and 198 mm^2^ for Q02, representing approximately 50% of the area of the dorsal part of central (0–7°) V1 (Figure S1). This was confirmed histologically in monkey Q02. Histological (Figure 1B) and radiological (Figure 1C) examination effectively ruled out the possibility that visually driven responses in areas V2, V3 might be due to surviving gray matter tissue within the area of the V1 lesion itself.

**Figure 2:**
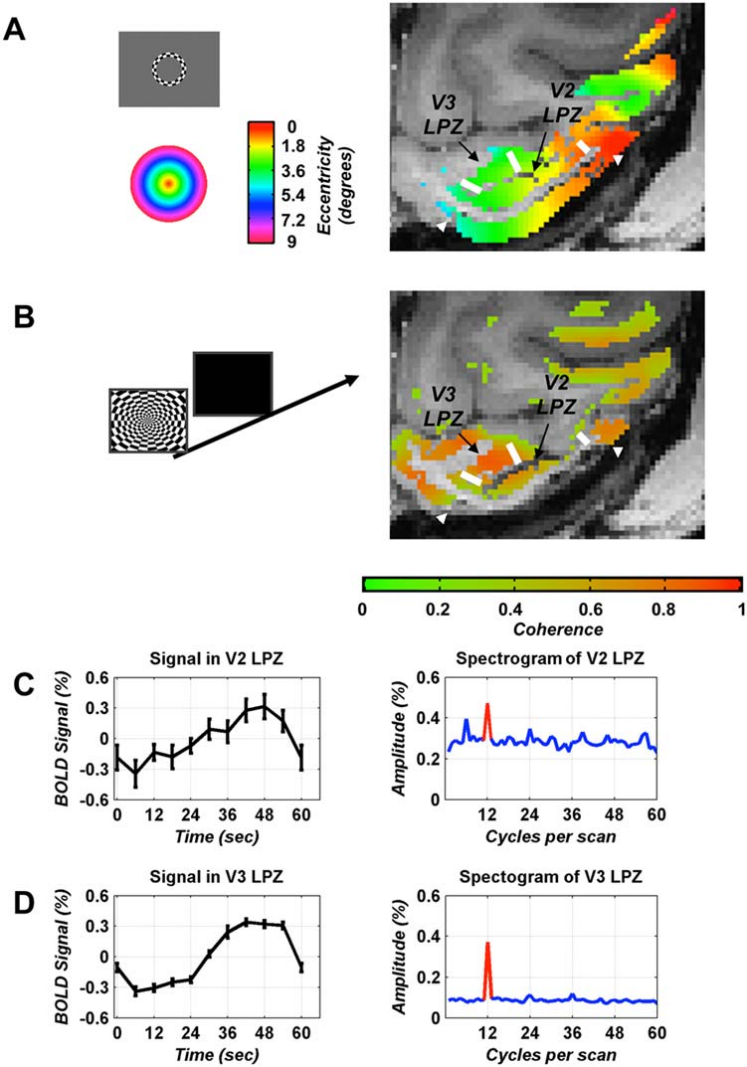
Visually elicited activation of the lesion projection zones (LPZ) in areas V2 and V3. A In a pre-lesion experiment, an expanding ring checkerboard stimulus (see Materials and Methods, gray inset) was used to obtain an eccentricity map of macaque visual cortex. Voxels within gray matter whose coherence was at least one standard deviation above noise level (coherence >0.5, see Materials and Methods) were color-coded according to the stimulus eccentricity that elicited the BOLD response (colored inset) and overlaid on anatomical images acquired after lesioning V1. An axial MR-slice through the early visual cortex of macaque L02 is shown here. For display purposes, the functional data were spatially smoothed with a 2D-Gaussian filter (FWHM = 2 mm), but this was not used in the analysis and does not affect the results. White arrowheads mark the border of the V1 lesion as seen on the anatomical scan. White bars mark the borders of the area V2, V3 LPZs. There is strong and retinotopically organized visually driven activity throughout areas V1, V2, V3 prior to the lesion. B To measure functional activation in the LPZ after lesioning V1, a full-field rotating checkerboard pattern alternating with periods of background illumination was used (data from monkey L02 obtained 188 days post-lesioning). Data display conventions are as in A, except now it is the value of the MR coherence measure that is color coded and overlaid on the anatomical slice. As expected, the area corresponding to the anatomical V1 lesion, outlined by the white arrowheads, is devoid of significant functional activation. However, surprisingly, visually driven activity is seen to persist inside the LPZs of both area V2 and V3. C Mean percent BOLD signal modulation (left) and mean amplitude spectrogram (right) over all voxels inside the area V2 LPZ. The stimulation frequency is displayed in red (12 cycles). Note that there is a significant stimulus driven BOLD modulation (∼0.6% peak to peak amplitude, z-score = 7.0). D Similar to C for the V3 LPZ (z-score = 33.3).

During the pre-lesion experiments visual stimulation with an expanding ring paradigm resulted in strong and reliable activation of the entire visual cortex (areas V1, V2, V3, V4, V5/MT) with single voxel coherence levels >0.7 and z-scores usually reaching values of >15 (Figure 2A). Specifically the gray matter in the part of area V1 to be lesioned showed reliable pre-lesion baseline activity (mean z-scores of 15.6 and 17.1 for monkeys L02 and Q02, respectively). Moreover, for both monkeys pre-lesion visually driven modulation was strong in the retinotopically corresponding areas of V2 (mean z-score 21.2 for L02, 17.5 for Q02) and V3 (20.4 for L02, 23.6 for, Q02), i.e. in the future lesion projection zones. The extent of the V1 lesion (Figure S1) and its effect on the activation of the LPZs in areas V2, V3 and on the activation of the non-lesioned part of V1 was assessed in each subsequent experiment, starting one month post lesion. As expected, strong activation of non-lesioned parts of V1 was preserved throughout the course of our experiments, whereas inside the lesioned V1 area coherence remained consistently at noise levels (∼0.37±0.13) for all post-lesion time points (Figure S1B). It is important to note that in both monkeys, the V1 lesion remained stable and inactive throughout the course of all experiments.

### BOLD-fMRI responses in areas V2 and V3

In the macaque monkey, the dorsal parts of areas V2 and V3 are located in the lunate sulcus [37], [38], [39], immediately anterior to dorsal V1, which provides their primary input. To examine the influence of the V1 lesion on V2 and V3 activity, we identified retinotopically the V1-lesion projection zones (LPZ) in these areas (i.e. we selected the regions in dorsal V2, V3 that in pre-lesion retinotopy contain the same eccentricity information as the V1 area to be lesioned). We confirmed the accuracy of the LPZ selections by measuring the distance of the defined V2, V3 LPZ borders from the foveal representation and comparing them to the predicted values derived from the extent of the V1 lesion and previously published magnification factors for V2 and V3 [37], [38]. Similar to the extent of the V1 lesion, the LPZs in V2 and V3 covered an area equal to ∼50% of the central (0–7°) dorsal V2 and V3 respectively. Figure 2A shows the baseline (pre-lesion) retinotopic eccentricity map for an axial slice through the macaque visual cortex overlaid on the corresponding anatomical slice taken after lesioning. Note the excellent correspondence between the eccentricities in the part of V1 to be lesioned (V1 region between the arrowheads) and the LPZ in area V2. The same holds for V3, though some phases are not seen in Figure 2A because they are out of the slice plane.

As expected, prior to lesioning the entire central sectors of areas V1, V2, and V3 were strongly activated by the retinotopic stimulus. This included the V1 area intended for lesioning (Figure 2A, between the white arrows) and its corresponding LPZs in areas V2 and V3. Figure 2B shows a map of coherence obtained using the full-field checkerboard stimulus approximately 6 months post-lesioning. As expected there is no visually driven activity inside the V1 lesion zone (outlined by the white arrows), since the grey matter is absent there. However, despite lack of V1 input significant visually driven BOLD signal modulation continues to be present inside the area V2, V3 lesion projection zones (outlined by the white bars). Figures 2C, D (left) plot the average time course of the mean BOLD signal, over all voxels inside the V2, V3 LPZ respectively. Stimulation is with the rotating full field checkerboard alternating with uniform background illumination. Note that despite the V1 lesion, significant visually driven modulation remains in both the V2 and V3 LPZ. This is reinforced by the mean amplitude spectrograms (Figure 2C, D – right), which show a large amplitude peak at the stimulation frequency (color red, 12 cycles per scan) that is significantly above noise (z-score = 7.0 and 33.3 for V2 and V3, respectively).

**Figure 3:**
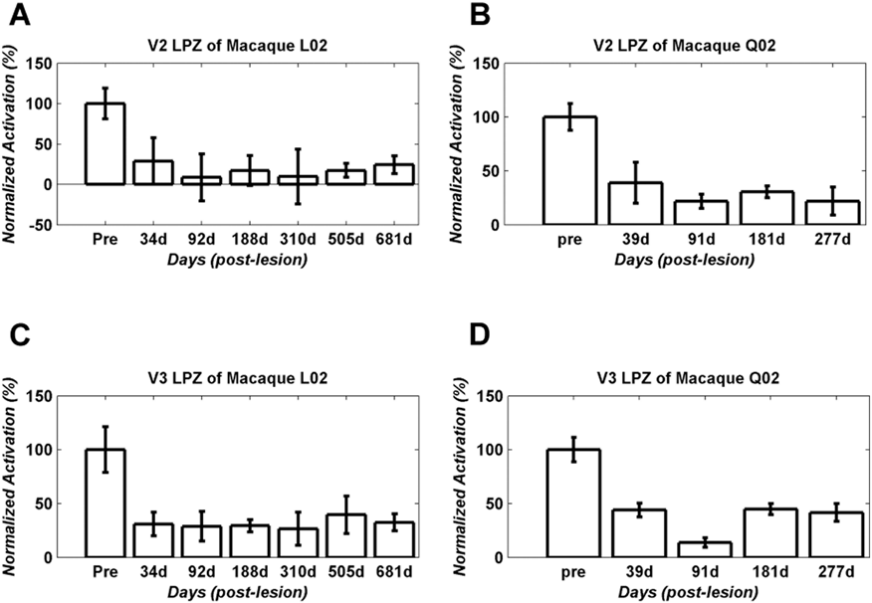
Analysis of changes in signal amplitude over time. BOLD activity (Mean±SEM) inside the LPZs of area V2 (upper panels) and V3 (lower panels) are plotted up to 681 days (L02, left panels) and 277 days (Q02, right panels) post-lesioning. To account for inevitable variability in the precise experimental/stimulation conditions across time points, BOLD signal strength is reported as percent modulation with respect to the corresponding cortical region in the unlesioned hemisphere, and is normalized by the pre-lesion value of this quantity (see Materials and Methods). Visually driven activity persists inside the LPZs of area V2 and V3 following the V1 lesions, though it is significantly reduced compared to pre-lesion levels. On average activity drops to ∼20–30% of pre-lesion levels. We observed no consistent increase of the visually driven activity over time from one month post-lesion until the last point checked (681 days).

We used the retinotopic stimulation paradigm to monitor the visually driven BOLD signal modulation strength in the LPZ of areas V2 and V3 following the V1 lesion and to compare it with pre-lesion values. Post lesion scans shown started one month following the lesion and proceeded up to ∼22.5, ∼9 months for monkeys L02, Q02 respectively. As can be seen from the post-lesion time points (Figure 3), although diminished, visually driven BOLD signal modulation remains significant inside the area V2, V3 lesion projection zones. Interestingly, activity levels did not change systematically over time during this observation period. We could therefore group together all time points following the lesion in estimating the change in BOLD modulation strength that occurs in the V2, V3 LPZ following the V1 lesion. We found that the mean percent BOLD modulation strength inside the area V2 LPZ dropped to 17.6±3.2% and 31.3±3.2% of its pre-lesion value for macaques L02 and Q02 respectively. The corresponding values for the area V3 LPZ were 28.3±4.1% and 36.0±7.5% for macaques L02 and Q02 respectively. Although clearly much weaker than pre-lesion levels, even this reduced modulation strength is surprising given the results from electrophysiology, which suggest that the vast majority (>95%) of V2, V3 neurons become inactive following transient V1 inactivation by cooling [1], [3]. There are several potential reasons for this difference which we will discuss in more detail below (see Discussion), including the different potential sources of the electrophysiological versus the BOLD signal, as well as the potential manifestation of cortical reorganization processes that are not present in the transient cooling studies. In any event, we can conclude that: 1) as expected, area V2, V3 activity is strongly dependent on area V1 input, yet 2) both areas V2 and V3 can be visually modulated even in the absence of retinotopically corresponding V1 input.

**Figure 4:**
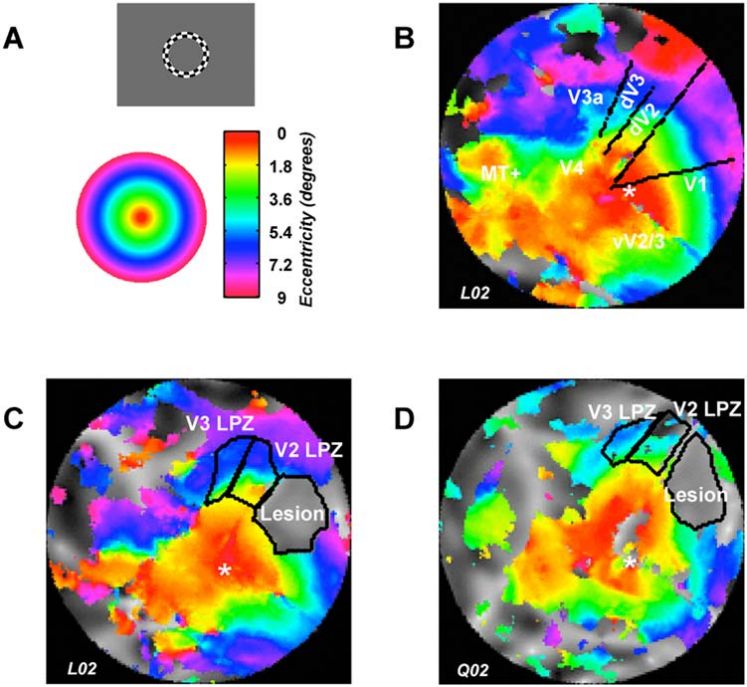
Eccentricity maps in non-lesioned versus lesioned cortex. A Expansion of the rotating checkerboard ring stimulus (gray inset) over time results in a phase shift of the BOLD response, which can be used to extract eccentricity information (Materials and Methods). Voxels with similar eccentricity information are color-coded (colored inset) and superimposed on the anatomical flat maps. B Organization of macaque L02's left, non-lesioned visual cortex 681 days post-lesion. For display purposes all data in this Figure have been spatially smoothed with a 2D-Gaussian filter (2 mm FWHM). Only voxels in gray matter with coherence exceeding one standard deviation above the single-voxel noise level of coherence (Materials and Methods) are displayed. This stimulus proved effective in evoking phase-locked responses from the fovea (red, * sign) up to about 9° (purple) eccentricities in V1, V2/V3, V3A, V4, and V5/MT+. Boundaries between dV1 dV2, dV3 and V3A were derived by mapping the visual meridians in independent experiments and are indicated here by the black lines. C Organization of macaque L02's lesioned (right) visual cortex 681 days post-lesion. Visual areas were identified as described in panel B. The lesion could be easily identified by the absence of gray matter as seen in a high resolution anatomical sequence MRI scan (Figure 1). It is located in the dorsal part of V1 representing the lower part of visual space in the contralateral quadrant and extending from ∼2 to 7° eccentricities. The lesion projection zones (LPZ) could be also easily identified in the dorsal parts of V2 and V3, using retinotopic correspondence criteria (Figure 2A, Materials and Methods). Note that despite the absence of direct retinotopically corresponding area V1 input, voxels in the LPZs of areas V2 and V3 exhibit retinotopically organized responses. D Organization of macaque Q02's lesioned (right) visual cortex 277 days post-lesioning. Extent of the lesion and retinotopically organized responses inside the LPZ of V2 and V3 are similar to monkey L02.

**Figure 5 pone-0005527-g005:**
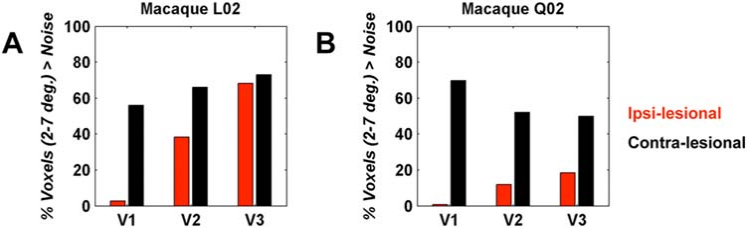
Percentage of supra-threshold voxels within 2–7° eccentricities across dorsal V1, V2, V3. A Data from macaque L02, 681 days post-lesioning. The bar height corresponds to the percentage of voxels within eccentricities 2–7° with coherence at least one standard deviation above the noise levels (Materials and Methods) relative to the total number of voxels sampled per area 0–7°. Black versus red bars correspond to data derived from the non-lesioned versus lesioned hemispheres, respectively. The effect of the lesion is manifest by the nearly complete absence of significantly activated voxels between eccentricities 2–7° in V1 of the lesioned hemisphere. The percentage of voxels within the same eccentricity range in areas V2 and V3 LPZs (red bars) remains below normal levels (black bars), but is markedly increased compared to the percentages measured in the lesioned part of V1. B Similar bar plot for macaque Q02, 188 days post-lesioning. Note that this animal had a weaker but still clear visual modulation response inside the V2, V3 LPZ.

To understand how the V2, V3 LPZ activity arises independent of retinotopically matching V1 input, we studied the eccentricity organization of the lesioned hemisphere and compared it to the non-lesioned hemisphere. We used the expanding ring stimulus paradigm (Figure 4A, Materials and Methods) to derive eccentricity maps in areas V1, V2, V3. The eccentricity maps of these areas in the non-lesioned hemisphere (Figure 4B) proved to be stable over time and remained commensurate to the maps obtained pre-lesion (Figure 2A), as well as to maps reported in the literature [40]. Figures 4C and 4D show the eccentricity map in the lesioned hemisphere of monkeys L02 and Q02 respectively, overlaid on the anatomical flat map (Materials and Methods). The lesion area has been defined anatomically by the absence of gray matter (as well as functional signal). Confirming the observation of a preserved cortical organization in a human blindsight subject [30], our data from V1 lesioned monkeys also demonstrate that the retinotopic structure of V1 cortex surrounding the lesion has not changed (Figure 4). Figures 4C,D show further that the BOLD signal inside the LPZs of both areas V2 and V3 remains retinotopically organized and, surprisingly, contains eccentricities corresponding to the lesioned V1 region (see also Figure 5). That is, eccentricity information not present in the lesioned portion of dorsal V1 (green and cyan phases corresponding to ∼3–5° eccentricity) is preserved inside the LPZs of areas V2 and V3 despite missing from their direct area V1 input. To further illustrate the relationship between V1, V2, and V3 in representing the visual field containing the lesion, we quantified the percentage of voxels between 2° and 7° with coherence >0.5 relative to the total number of voxels per area and compared these measures for the lesion-ipsilateral (red bars) and lesion-contralateral (black bars) hemispheres (Figure 5). Across monkeys and visual areas between 50 and 75% of the voxels representing the visual space between 2° and 7° exceeded coherence >0.5 in the non-lesioned hemisphere. The lesion in V1 resulted in the virtual absence (<3%) of supra-threshold voxels in this part of the visual field. Yet despite lacking direct dorsal V1 input from within this eccentricity range, 12 to 68% of voxels between 2° and 7° in areas V2 and V3 reached coherence >0.5. Therefore, information about the visual world not represented in the preserved portion of dorsal area V1, nevertheless reaches dorsal areas V2 and V3. This suggests that: 1) the activity observed inside the area V2, V3 LPZs cannot arise as a result of input arising from dorsal V1 locations that lie outside the lesion (as might occur if the observed activity were due to a subpopulation of V2, V3 cells with large receptive fields reaching outside the lesioned area), and 2) the observed visual modulation in the LPZ of areas V2, V3 must be driven by retinotopically organized cortical or subcortical areas.

**Figure 6:**
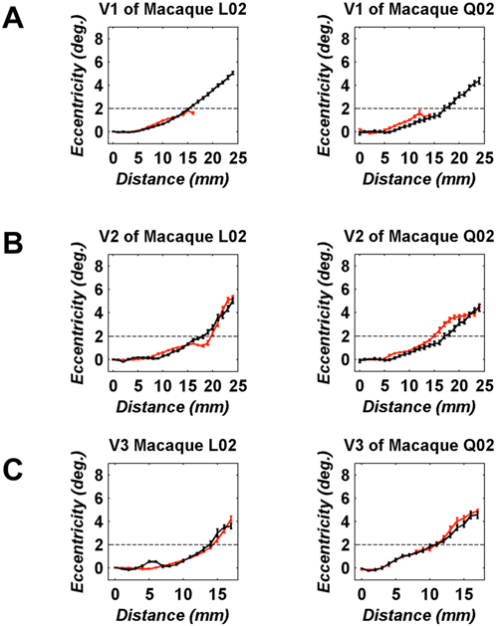
Eccentricity versus cortical distance plotted across areas V1, V2, V3, in both the lesioned (red lines) and the non-lesioned (black lines) hemisphere. Data were obtained from macaques L02, 681 days (left panels) and Q02, 188 days (right panels) post-lesioning. A Eccentricity versus distance curves in dorsal area V1. The dashed line indicates the foveal border of the lesion. In both monkeys, beyond ∼2° no eccentricity information is present in dorsal V1. Data represent Mean+/−SEM from 10 iso-angle, radial ROIs (Materials and Methods). B Eccentricity versus distance curves in area V2. Conventions are as in (A). Data represent Mean+/−SEM from 10 iso-angle radial ROIs. C Eccentricity versus distance curves in area V3. Data represent Mean+/−SEM from 4 iso-angle, radial ROIs. Note that the V1-Lesion projection zones in areas V2, V3 retain the representation of eccentricities that are not present in their dorsal V1 input.

To investigate whether the retinotopic organization of areas V1, V2, V3 is changed (e.g. reorganized) after V1 lesioning, we compared visual eccentricity versus cortical distance plots (Materials and Methods) across these areas both in the lesioned and the intact hemispheres. Figure 6A shows these plots in area V1 for monkeys L02 and Q02. The V1 lesion starts at the dashed lines (>2° eccentricity), and the color of the graph denotes whether the plot is derived from the lesioned (red) or the non-lesioned (black) hemisphere. There is excellent overlap of the eccentricity versus distance curves in foveal V1, outside the area of the lesion, suggesting that the magnification factor remains unchanged in nearby V1 cortex following the lesion. As in area V1 little, if any, magnification factor change is seen outside the LPZs in areas V2, V3. Overall, the eccentricity versus distance plots in area V2, V3 of the lesioned hemisphere follow the trend seen in the intact hemisphere, though small but significant deviations are noted from the expected pattern inside the region of the LPZs (Figure 6B). Note that although eccentricity information arising beyond ∼2° is absent from dorsal area V1 after the lesion (Figure 6A), it nevertheless persists in the LPZ of areas V2, V3 (Figure 6B, 6C). This suggests that the observed responses are mediated via retinotopically organized subcortical or cortico-cortical (feedback or callosal) connections, which continue to have access to the appropriate eccentricity information.

**Figure 7:**
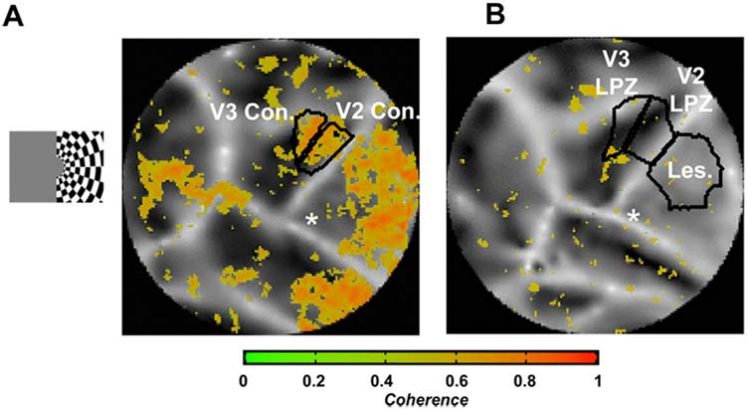
Activation maps obtained by restricting the stimulus to the unaffected (ipsilateral to the lesion) visual field. A rotating checkerboard stimulus extending from 3° to 13° was presented for 30 seconds in the ipsi-lesional visual field alternating with 30 second periods of uniform illumination. The data presented here were obtained from one scan in monkey L02. A Functional activation map of the left, non-lesioned hemisphere. Note that the stimulus was effective in evoking significant activity in the retinotopically matched control areas corresponding to the area V2, V3 LPZs. B Activation map of the right, lesioned hemisphere. The stimulus did not evoke significant visual modulation responses inside the LPZs of areas V2 and V3.

In an fMRI study on human blindsight subjects, Goebel et al. reported that responses in areas V4 and V5/MT of the lesioned (but not the non-lesioned) hemisphere could be driven by stimulation in the ipsi-lesional visual field [29], which suggests that callosal input may play a role. However, callosal input is not likely to play a major role in our study since in areas V2, V3 it tends to concentrate near the vertical meridians [39], [41], [42] while the responses displayed in Figure 4 appear to occur over the entire V2, V3 LPZ regions spanning the cortex from vertical to horizontal meridians. In addition, visual stimulation restricted to the ipsi-lesional visual field (visual field projecting to the intact hemisphere) did not evoke significant activity inside the area V2, V3 LPZs, despite being able to drive retinotopically corresponding regions in the intact hemisphere (Figure 7) This strongly suggests that callosal input does not play a critical role in the generation of the observed patterns of activity.

The amplitude of visually driven BOLD signal modulation inside the V2, V3 LPZ (20–30% of pre-lesion levels) is surprising in light of transient inactivation studies [1], [2], [3], which report that neuronal activity is suppressed in more than 95% of sampled V2, V3 sites. Since, under usual stimulation conditions the BOLD signal correlates well with multi-unit activity in the neocortex [32], [33], [43], [44], [45], [46], one might have expected the strength of the BOLD signal modulation inside the V2, V3 LPZ to fall to less than 5% of its pre-lesion value, as opposed to the ∼20–30% we report here. However, the BOLD signal does not simply reflect multi-unit activity, but it also depends on sub-threshold synaptic currents, as reflected in the local field potential [32]. It therefore remains possible that the BOLD signal responses we observe here may in part reflect subthreshold synaptic events, not directly reflected in the recordings of prior transient inactivation studies [1], [2], [3].

### Multi-unit activity in area V2

**Figure 8:**
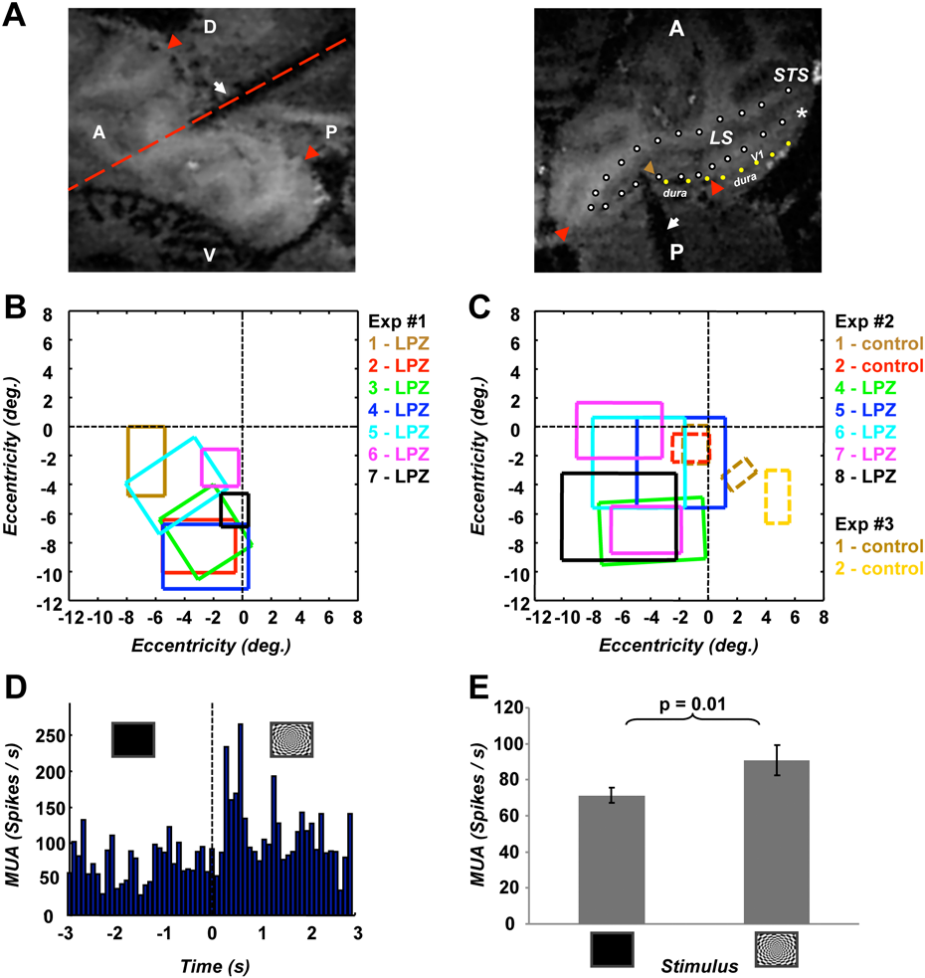
Electrophysiological measurements inside the V2 LPZ. A The left panel shows a parasagittal slice of an anatomical MR scan of macaque L02. The red arrows show the borders of the V1 lesion in this plane. The red-dashed line represents the position of the image plane shown on the right. In the right panel, the recording electrode can be seen to penetrate the lesioned portion of area V1 and lie with its tip (brown arrow) located in V2 near the fundus of the lunate sulcus (LS), where the horizontal meridian representation separates areas V2 and V3. The red arrow tips mark the boundary of the V1 lesion in this plane (from ∼2^0^ eccentricity to near the midline, see Figure 2). The white dotted line outlines the cortical gray matter located in the lunate sulcus, whose posterior bank corresponds to area V2. The yellow dotted line outlines the dural boundary. Note that the yellow and the white dotted line come together near the lateral red arrow, marking the end of V1 gray matter and the beginning of the lesion. During the penetration shown, multi-unit activity (MUA) was recorded from 4 different electrode positions (positions 1 to 4 shown in panel b). Recordings were first performed in position 1 (brown arrow in the lunate fundus) and then the electrode was stepwise withdrawn 500 µm at a time, until it exited gray matter (total distance travelled ∼2 mm). Positions 2, 3, and 4 traverse the posterior bank of the lunate, positioned solidly inside the V2 LPZ. Retracting the electrode beyond position 4 led it to exit gray matter, a further confirmation that the corresponding portion of V1 had been completely lesioned (this has also been visualized anatomically with a high resolution MR image, and confirmed functionally –see Figure 1C, 2B, 4C). (*) marks the foveal representation in V1. LS = Lunate Sulcus, STS = Superior Temporal Sulcus, A = Anterior, P = Posterior, D = Dorsal, V = Ventral. B Receptive field (RF) maps of multi-unit activity (MUA) recorded during the penetrations illustrated in panel A, (positions 1–4 in panel B), and during two separate, more lateral penetrations inside the V2 LPZ (receptive fields 5, 6). RFs were mapped manually using small oriented bars (width×length = 0.5^0^×1^0^); see Materials and Methods). Note that electrode location #1 yields a receptive field map close to the horizontal meridian (as expected from the electrode position in the lunate fundus near the border between V2, V3). Locations 2,3,4 of the same tract were situated squarely in area V2, on the posterior bank of the lunate sulcus and the corresponding multi-unit receptive fields lie closer to the vertical meridian. Note that receptive fields overlap with the LPZ of fMRI maps (Figures 4 & 6), but are not necessarily confined to it. Importantly, responses could be elicited even when visual stimulation was entirely restricted within the scotoma confirming that they arise via a subcortical pathway that bypasses area V1. C Multi-unit RF-plot obtained from a linear array of 8 electrodes during a separate experiment from the same V1-lesioned animal as in panel B. Electrode number 3 broke on entry and recorded no multi-unit activity. Multi unit receptive fields from the interior of the LPZ (sites 4–8) are represented by solid lines, whereas receptive fields obtained outside the LPZ, in non-deafferented V2, by dashed lines (control sites 1,2). Two multi-unit receptive fields illustrated in the right inferior quadrant were obtained from a monkey without a V1 lesion (control exp #3). Note that multi unit receptive fields obtained inside the V2 LPZ are much larger than those obtained at similar eccentricities in non-deafferented V2 cortex, and on one occasion (electrode #7) bipartite. Receptive fields obtained at a second cortical depth (500 µm away) for each electrode penetration were found to be commensurate. Here only one set is illustrated. D Multi unit activity peri-stimulus-time-histogram (PSTH) centered at the onset of full-field visual stimulation (Materials and Methods) recorded from electrode position 5 (panel B). The signal was high-pass filtered at 312 Hz and thresholded at 3 standard deviations beyond the mean. Each bar height corresponds to the spike rate calculated within 100 ms bins, and averaged over 10 stimulation cycles. The multi-unit firing rate at this position increases by more than 50% when the stimulus is on. E Summary of multi unit activity elicited from all recording locations shown in panels B and C inside the V2 LPZ. To elicit visual responses under the same conditions as in the fMRI experiments (Figure 2 B), the same full field rotating checkerboard stimulus was alternated with periods of background illumination. On average, MUA increased by ∼30% during visual stimulation (p = 0.01, one-tailed paired-samples t-test) compared to baseline.

To directly confirm that the observed BOLD signal responses accurately reflect modulation in the underlying multi-unit spiking activity we performed two electrophysiology experiments in the one remaining animal L02 (monkey Q02 was euthanized for histological evaluation of the lesion). The position of the electrode in the posterior bank of the lunate sulcus (LS) was directly monitored in relation to the V2 LPZ by MRI. In the first experiment, we recorded from 7 sites along 3 different electrode tracks (Figure 8A, B), all located deep in the interior of the V2 LPZ. In the second experiment (Figure 8C) we recorded from 7 cortical locations spanning the V2 LPZ border (5 inside, 2 outside the V2 LPZ), at two cortical depths per electrode (one superficial, one deep separated by ∼500 µm). Two additional sites, two depths each, were recorded from the V2 of a non-lesioned animal at eccentricities commensurate to the V2 LPZ (Figure 8C, right lower quadrant, Exp #3). Remarkably, multi- unit activity minimal response fields could be mapped manually at each site inside the V2 LPZ (Figure 8B, C), using small (0.5^0^×1^0^) bar stimuli at different orientations flashing or moving perpendicular to their axis. There receptive fields were more weakly driven but much larger than corresponding receptive fields obtained in non-deafferented V2: for example, receptive field #4 (8B) is approximately 6^0^×5^0^, while receptive field #7 (8C) is not only large but bipartite. It is important to point-out that visual responses could be elicited even when the stimulus lay entirely inside the scotoma induced by the V1 lesion, strongly suggesting that activity inside the V2 LPZ arises through subcortical channels that bypass the V1 input. We also measured the multi-unit activity modulation elicited by the full field (26^0^×20^0^) checkerboard stimulation we used in the fMRI experiments (Materials and Methods). Figure 8D plots the mean multi-unit activity modulation at one representative penetration in the interior of the V2 LPZ. The amplitude of the mean visually driven multi-unit activity modulation across all recording sites in the interior of the LPZ (17 sites from two experiments) was ∼30% (p = 0.01, one tailed, paired samples t-test) compared to baseline (Figure 8E). It is then clear that the difference between our observations and the results of transient inactivation studies based on electrophysiology [1], [2], [3] cannot be discounted based on the BOLD signal's sensitivity to subthreshold synaptic activity.

In summary, our results confirmed that activity in areas V2 and V3 is strongly dependent on input from area V1. Despite this however, both areas V2 and V3 show significant visual modulation following the chronic absence of retinotopically matched V1 input. This contrasts with reports from transient V1 inactivation studies [1], [2], [3], suggesting that visually driven activity inside the V2, V3 LPZ may be the result of neural reorganization (for example increased gain control in subcortical pathways projecting to areas V2, V3) following chronic V1 lesions. If so, this likely occurs within the first month post-lesion since BOLD signal modulation strength does not change systematically from 1 to 22 months post lesion. Surprisingly, V1-lesion projection zones in areas V2, V3 retain the representation of eccentricities eliminated from their direct V1 input. Inputs from the intact dorsal V1-lesion surround or from the contralateral hemisphere through the corpus callosum do not appear to mediate the residual responses.

## Discussion

We monitored visually driven responses in macaque areas V2 and V3 over several months following the induction of area V1 lesions. In contrast to previous studies that examined single- or multi-unit activity in dorsal V2, V3 following reversible cooling of V1 [1], [3], [25], we found that visually driven activity in V2 and V3 deprived of input from retinotopically corresponding V1 locations was not completely silenced, though it dropped to ∼20–30% of pre-lesion levels. This level of activation did not change systematically during the period examined here (1 to 22 months post-lesioning). Interestingly, in spite of the observed reduction in BOLD response amplitude, the retinotopic organization of areas V2, V3 remained similar to before the lesion. Electrophysiological recordings of multi-unit activity inside the V2 LPZ corroborated the return of significant visual modulation that was observed using the BOLD signal. In what follows, we discuss our results in the context of the existing literature and speculate on what pathways mediate the activity that persists following the V1 lesions.

### V2 and V3 activity in the absence of V1 input

The persistence of V2 and V3 responses despite missing V1 input may not be surprising from an anatomical point of view: There are several V1-bypassing connections to macaque areas V2 and V3 which originate in subcortical (for example: LGN or inferior and lateral Pulvinar nuclei) as well as cortical (for example: callosal input, V4 or V5/MT feedback) structures [41], [42], [47], [48], [49], [50], [51], [52], [53], [54], [55], [56]. Nevertheless, studies in which single- or multi-unit activity was recorded in the minutes following transient inactivation of V1 by cooling report that >95% of V2, V3 sites stop responding entirely to the visual stimulus [1], [3], [25]. This contrasts with the 20–30% residual BOLD signal amplitude modulation we report here. Electrophysiology experiments in one animal corroborated the fMRI results, confirming a different picture than transient inactivation studies: i) all 17 multi-unit sites recorded inside the V2 LPZ could be visually modulated using small (0.5^0^×1^0^) oriented bar stimuli, and ii) the resulting multi-unit receptive fields were all unexpectedly large, and in one occasion bipartite (Figure 8). While these results seem at odds with earlier studies of V2 and V3 responses following transient V1 inactivation [1], [3], [25], similar results have been obtained for area V5/MT [19], [22]. There are at least two factors that may explain the differences between the earlier V2/V3 findings and the results of the present study:

1. The positive V2/V3 responses we report here were first observed 1 month after lesioning area V1 whereas the earlier studies report V2/V3 responses immediately (minutes to hours) after V1 cooling [1], [3], [25]. Therefore the stronger V2/V3 activation observed in our study may reflect a degree of reorganization occurring within the first month following the V1 lesion (see also Figure S2). This postulated reorganization might be due to a strengthening of V1-bypassing inputs (e.g. from the thalamus or the Pulvinar) from modulatory to driving. Early reorganization notwithstanding, our data argue that little if any spontaneous reorganization occurs after one month post lesion: Specifically, we did not observe a significant systematic increase in the strength of the visual responses recorded inside the lesion projection zones of neither V2 nor V3, from 1 month up to 22 months post-lesioning (Figure 3). Moreover, during this time, there was no systematic change in the eccentricity versus cortical distance curves of the lesioned compared to the non-lesioned hemisphere, which is another way that plasticity may manifest [23], [57]. 2. On a more technical note, it is possible that the cooling inactivation applied in the earlier electrophysiological studies may have inadvertently inactivated V1-bypassing fiber connections coming to V2/V3 from subcortical structures, which might in effect have resulted in double de-afferentiation of these areas and may have contributed to rendering V2, V3 cells unresponsive. Although our lesioning method (aspiration) may also in principle undercut direct inputs to V2, V3 this would tend to decrease V2, V3 responses, rendering the measurements we report here an underestimate of the true visual modulation strength.

### Flow of visual information in the absence of V1

Human patients and monkeys with area V1 lesions, despite reporting being blind within the affected part of the visual field, can nevertheless retain certain visual capacity under specific conditions (“blindsight”) [6], [58]. This raises the question of how such “blindsight” might be mediated in the brain. One step in trying to map potential neural pathways that may mediate various aspects of the phenomenon of blindsight is to study the patterns of extrastriate cortical activity in the absence of V1 input, as was done here. Although the prevalent view is that blindsight may be mediated by activity in higher cortical areas such as V5/MT [27], [28], [29], it is clear from the data we present here that, visually driven activity in the absence of V1 input also persists in early extrastriate areas V2 and V3, which may therefore contribute to this phenomenon.

Although our data strongly argue that areas V2 and V3 are capable of contributing to V1 independent visual processing, the specific pathways that mediate the V1-independent responses in these areas remain unclear. One possibility is that the residual visual modulation is mediated via callosal connections [41], [42], [56] from the non-lesioned hemisphere. However, activity in the V2 and V3 LPZs could not be evoked when restriciting visual stimulation to the intact visual hemifield (Figure 7). Thus it appears that activity in early extrastriate cortex devoid of V1 input must come about through V1-bypassing subcortical channels, potentially also involving feedback from higher areas. The strongest argument that V1-bypassing subcortical channels must be involved in generating the observed V2, V3 activity is provided by multi-unit receptive field maps recorded inside the V2 LPZ (Figure 8). Specifically, multi-unit activity at recorded V2 sites was clearly modulated by the visual stimulus (0.5^0^×1^0^ oriented bars) even when the latter was entirely contained within the scotoma corresponding to the V1 lesion.

The superior colliculus receives input from ∼10% of the retinal ganglion cells in its superficial layers [59], and has been shown to be important for mediating activation in extrastriate cortex. Residual responses observed in V5/MT [60] and STP [26] following V1 lesions could be completely silenced by lesioning the ipsilateral superior colliculus. The lateral geniculate nucleus (LGN) and the Pulvinar both receive collicular input and show retinotopic organization [61], [62]. LGN cells project directly to areas V2, V4, and V5/MT [51], [52], [54], [63]. Specifically for area V2, recent evidence suggests that projecting cells in the LGN are primarily located in the intercalated layers, in which cells of the koniocellular type predominate [51], [63] and which receive at least part of their input from the superficial layers of the superior colliculus [64]. Alternatively, or perhaps additionally, residual extra-striate activity could be mediated through the Pulvinar. The importance of the Pulvinar for extrastriate cortex activation is still not entirely clear, yet anatomical projections from the Pulvinar to areas V2, V3, V3A, V4, V5/MT, and V5/MT [47], [48], [65], [66] suggest that it plays a potentially important role in visual processing. With respect to V2 and V3, not much is known, though it has been shown that the responses of V2 neurons can be modulated by input from the Pulvinar [67]. It remains to be seen however whether Pulvinar input is capable of activating area V2, V3 neurons in the absence of V1 input.

An important question that remains is whether higher cortical areas, particularly those with fast response latencies (such as V5/MT or FEF) play a role in generating the persisting activity in areas V2, V3 after V1 lesions. For example, activation of area V5/MT mediated by superior collicular inputs [60] through the Pulvinar [68] or the LGN might, through existing feedback projections to V2 and V3 [49], [53], generate the activity patterns we observe there. Such a scenario is theoretically possible also for area V4 given the existence of feedforward anatomic connections from LGN [54] and Pulvinar [47], [65] to this area, as well as feedback connections from V4 to V2, V3 [50], [55]. At present we cannot definitively distinguish between i) direct, V1-bypassing, subcortical inputs to V2, V3 versus ii) indirect, V1-bypassing, subcortical inputs reaching V2, V3 via feedback pathways from higher areas, as a source of the observed activity.

Finally, we would like to note that time elapsed following the lesion may turn out to be of critical essence: Although our results suggest that activation levels in V2 and V3 remain stable from 1 to 22 months post-lesioning, when compared to the results of transient inactivation studies [1], [3], [25] they also suggest that plasticity processes operating within the first month post lesion likely play a role in modulating the gain of cortical networks responsible for the observed activity. Furthermore, these processes may be strongly dependent on behavioral training [8], [10], which we did not explore here.

In summary, our results reveal that V2 and V3 can process visual information in the absence of retinotopically corresponding V1 input, and point to V1-bypassing subcortical structures as the likely pathway for generating the persisting patterns of activity in early extrastriate cortex. Defining the precise pathways involved and how plastic or amenable to behavioral modification they are will be the aim of future studies. The cortical networks mediating the phenomenon of blindsight remain to a large extent a mystery to date, but our results lend credence to the belief that areas as early as V2 and V3 may potentially contribute to this phenomenon. Finally our study demonstrates the potential promise of macaque fMRI for the serial, non-invasive, in-vivo, monitoring of cortical organization following controlled nervous system injury.

## Materials and Methods

### Mri data collection

Measurements were made on a vertical 4.7 T scanner with a 40 cm diameter bore (Biospec 47/40v, Bruker Medical, Ettlingen, Germany). The system was equipped with a 50 mT/m (180 ms rise time) actively shielded gradient coil (Bruker, B-GA 26) of 85 mm inner diameter. A radiofrequency coil with an inner diameter of 85 mm was placed over the monkey's occiput to acquire images from visual cortex. To optimize homogeneity of the MR signal from the visual cortex, Fastmap-shimming [69] of this area was performed using an 18×18×18 mm^3^ box. Acquisition of functional data was performed using 8-shot gradient-recalled EPI [70], [71] with a voxel resolution of 1×1×2 mm^3^ (17 slices, FOV = 128 mm×128 mm, Matrix = 128×128, TR = 8 shots×750 ms, TE = 20 ms, FA = 40 deg). Within-session anatomical images (0.5×0.5×2 mm^3^ resolution) were acquired using either IR-Rare (Hahn Spin-Echo with Rare-Factor) or Mdeft sequences [35]. Whole-brain anatomical images were acquired using the 3D-Mdeft sequence [34] (128 slices, 0.5×0.5×0.5 mm^3^ resolution) and were used for co-registering data from different experiments.

### Animal Preparation

Two healthy adult macaca mulatta (Q02, L02), older than 4 years (weight: 6–8 kg), were used for the experiments. All sessions were in full compliance with the guidelines of the European community for the care and use of the laboratory animals (EUVD 86/609/EEC) and were approved by the local authorities (Regierungspräsidium).

Surgical operations were performed following standard surgical procedures described elsewhere [31]. To lesion V1, the skull was opened under sterile conditions over the occipital gyrus, and the dura was reflected. Pial vessels over the intended lesion area were coagulated and low suction aspiration was applied via a 20 gauge catheter until white matter was reached (∼1.8 mm from the cortical surface). The lesions reached medially to within ∼2 mm of the internal calcarine sulcus, dorsally to ∼2 mm from the lunate sulcus, and exended ventrally to include the external calcarine fissure. The extent of the lesions was 13×17 mm (monkey Q02) and 16×17 mm (monkey L02) along the medial to lateral and dorsal to ventral axes, respectively. After waiting for hemostasis to set in, the dural flap was sutured back in place. Finally the bone flap was put in place and secured via sutures and Calcibon® (Biomet, Merck, Berlin, Germany).

FMRI experiments were done under general anesthesia according to previously published protocols [31], [33]. In brief, the animal was intubated after induction with fentanyl (31 µg/kg), thiopental (5 mg/kg) and succinylcholine chloride (3 mg/kg) and anesthesia was maintained with remifentanyl (35 µg/kg/h). Mivacurium chloride (6 mg/kg/h) was used after induction to ensure complete paralysis of the eye muscles. Lactate Ringers with 2.5% glucose was infused at 30 ml/h. For monkey L02 10 mg Phenylephrine were diluted in 500 ml Lactate Ringer and dialled at ∼10 ml/h (0.2 mg/h) to maintain systolic blood pressure at constant levels (Mean±Std for systolic/diastolic: 101±12/54±9) throughout the experiment. The temperature was maintained at 38–39.5 C°. After achieving mydriasis with two drops of 1% cyclopentolate hydrochloride, each eye was fitted with hard contact lenses (Harte PMMA-Linsen Firma Wöhlk, Kiel, Germany) to bring it to focus on the stimulus plane (∼2 diopters).

Following the end of the fMRI experiments animal Q02 was sacrificed by a lethal injection (60 mg/kg) of Pentobarbibal (the second animal (L02) is still involved in follow up experiments so the extent of its lesion was confirmed radiologically). After transcardial in vivo perfusion (0.4% paraformaldehyde pH 7.4, in 0.1 M phosphate buffer solution), the brain was quickly removed and placed in 30% sucrose solution in phosphate buffer. 100 µm thick sections were cut on a cryotome, mounted on slides and dried overnight at 39°C. Sections were then stained in Cresyl Violet solution for 1–2 min and dehydrated in a series of ethanol (50–100%), Butanol and Xylene for 5 minutes each. Finally they were coverslipped with DePex (SERVA, Heidelberg, Germany) and studied under a microscope.

Two electrophysiology experiments were performed, under anesthesia, on animal L02 at the end of the fMRI experiments (>2 years post-lesioning). An MR-compatible recording chamber made of PEEK was implanted over the monkey's primary visual cortex to overlap extensively with the V1 lesion. In experiment 1, electrodes (Platinum-Iridium, 1–2 MΩ impedance), were lowered under direct MRI visualization to reach the interior of the V2 LPZ in the posterior bank of the lunate sulcus. During this experiment, recordings were made from 7 different sites with 3 different recording tracks. In a second experiment a linear array of 8 electrodes (Tungsten, 1–2 MΩ impedance), spaced at 1 mm intervals, was placed across the LPZ border through a recording grid whose positioning was later confirmed in a separate MRI session. Two cortical depths (one superficial, one 500 µm deeper) were mapped per penetration. Receptive field maps at different depths were commensurate.

### Visual stimulation

Visual stimuli were presented using an SVGA fiber-optic system (AVOTEC, Silent Vision) with 640×480 resolution and a 60 Hz frame rate. The field of view was 26° horizontal×20° vertical visual angle. All stimulation was done monocularly. Care was taken to align the center of the stimulus to the fovea.

The basic stimulus pattern used in all experiments consisted of a polar checkerboard pattern (∼3.5 Hz visual modulation at 100% contrast). The direction of rotation reversed every 1.5 sec to avoid adaptation. A full-field (26° horizontal×20° vertical) version of this stimulus (30 s presentation) alternating with periods of uniform illumination (30 s) was used for the data presented in Figure 2B. Retinotopic mapping was done using the expanding rings paradigm [40], [72]: We used annuli with a width of 1.5° whose outer radius expanded from 1.5° to 9° (11 annuli) in steps of 0.75° (Figure 2A). Each annulus was presented for 6.0 s ( = 1 TR) and the entire sequence of annuli was repeated 12 times per scan. This stimulus paradigm has been shown to activate well early visual areas in the macaque [33], [40]. For the data presented in Figure 7, the checkerboard stimulus extended from 3° to 13° and was restricted to the intact (ipsilateral to the lesion) visual field. This stimulus was presented for 30 s alternating with 30 s periods of uniform intensity (background) illumination.

For electrophysiological recordings, minimal multi-unit response fields were mapped manually using oriented bar stimuli (width×length = 0.5^0^×1^0^). Mapping was performed after waiting for 30–60 minutes for the signal to stabilize. Finally we also applied the same full-field checkerboard stimulation paradigm as during the fMRI-sessions (see Materials and Methods), albeit with shorter (10 s) stimulation (ON) and background (OFF) periods.

### Data analysis

We used the mrVista software (http://white.stanford.edu/software) for data analysis. In a typical experiment 5 to 10 repeats of the expanding ring stimulation paradigm were performed and the average BOLD signal time course was generated. Using the average time course, we then assessed the strength of the BOLD signal in each voxel independently by using the measure of coherence [33], [40], [72]:
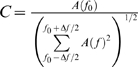
, where f_0_ is the stimulation frequency, A(f_0_) the amplitude of the BOLD signal at that frequency, and Δf (noiseband) a range of frequencies around the fundamental (f_0_). For the stimuli that we used, f_0_ corresponded to 12 cycles per scan and Δf was chosen to be a bandwidth of 6 cycles (that is from 9 to 15 cycles) centered at f_0_. Note that the noise level of coherence depends on the chosen bandwidth. So that for Δf = 6 cycles under the assumption of a white noise distribution, the noise level of coherence (the coherence value in the absence of visual modulation) is expected to be 1/(Δf+1)^0.5^ = 0.38. In agreement with this estimate, the noise level measured in a rectangular ROI outside the brain, in muscle, or in non-stimulated parts of cortex reached a coherence level of 0.37±0.13 (mean±std). This noise level proved to be stable across monkeys and experiments.

Functional activation maps were plotted using a coherence threshold of 0.5 corresponding to 1 standard deviation above the mean single-voxel noise level, and were co-registered with and overlaid on high-resolution anatomical maps. To visualize activity located in cortical sulci, cortical flat maps were constructed by using the mrGray software to segment gray/white matter and flatten the visual cortex [73]. The functional activity of gray matter voxels was overlaid onto the flat maps. For display purposes (but not for the quantitative analysis), functional data shown in Figures 2, 4, and 6 have also been smoothed with a 2D-Gaussian spatial filter (FWHM = 2 mm, i.e. 2 voxels).

In order to quantify the strength of visual stimulation inside the area V2, V3 LPZs (Figure 3), the amplitude of the mean BOLD signal over the LPZ at the stimulation frequency (f_0_) minus the mean amplitude in the noiseband (excluding the stimulation frequency) was normalized using a retinotopically matched control ROI in the intact hemisphere:
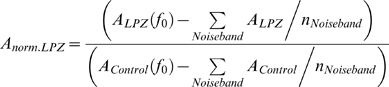



In the expanding ring paradigm the visual stimulus is displayed in different spatial locations over time which translates into a BOLD signal phase shift in correspondingly activated cortical voxels. Information about the maximum stimulus expansion (9°) in one cycle of stimulation (2π) was used to convert the radian phase value (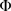) of each voxel into the corresponding eccentricity information:
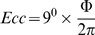



The eccentricity information over all active voxels could then be used to plot the representation of eccentricity information in visual cortex as a function of distance (Figures 2, 4–6). To plot the eccentricity versus cortical distance functions (Figure 6) for visual areas V1, V2, and V3, we selected from the smoothed flat maps (Figure 4) 4 (V3) or 10 (V1, V2) iso-angle, radial, ROIs extending from the fovea to 4–5° eccentricity near the center of the lesion, and sorted the contributing voxels according to their distance from the foveal representation.

To measure multi-unit activity (MUA) the recorded signal was high-pass filtered at 312 Hz and thresholded at 3 standard deviations beyond the mean. Spike rates were measured in bins of 100 ms (Figure 8D). To compare the mean MUA between stimulus (ON) versus background (OFF) periods (Figure 8E), for each experimental run the spike rate 3 seconds before and after stimulus onset were averaged across 10 stimulation cycles.

## Supporting Information

Figure S1 Stability of the V1 lesion. The V1 lesion could be easily identified in each experiment from the anatomical (MDEFT) MR images by the absence of gray matter. A..The number of voxels defining the lesion was plotted over time for monkeys L02 (left) and Q02 (right). B. The mean coherence over all voxels within the lesion was plotted over time for monkeys L02 (left) and Q02 (right). The dashed line corresponds to a coherence level of 1 std>mean noise levels (see methods). The lesion remained stable and inactive over the entire time period.
(0.14 MB TIF)

Figure S2 A Comparison of fMRI activity hours versus 39 days post-lesioning. A representative axial slice from the macaque visual cortex with overlaid coherence (left column) and phase (right column) activation maps. The top row represents data obtained on day 1 (∼12 hours after the V1 aspiration lesion), the second row data obtained on day 39. In this slice, the lesion spares a small portion of V1 near the lunate sulcus (which explains the small focus of activity seen outside the foveal border of the V2 LPZ in the upper panels) and extends medially nearly to the midline. Note that on day #1 the V2 LPZ is not significantly modulated by the visual stimulus, in contrast to the strong area V2 modulation seen in the contralateral (intact hemisphere). By day 39, it is clear that weak but significant modulation has returned to the V2 LPZ. B Mean percent signal modulation inside the V2 LPZ during presentation of a ring stimulus centered at 4°. Signal modulation, absent at the first day post-lesioning (red line), recovers within 39 days post-lesioning (black line).
(0.26 MB TIF)
