# Supplementary figures and images for: Visually Driven Activation in Macaque Areas V2 and V3 without Input from the Primary Visual Cortex
Source: PLoS One. 2009 May 13;4(5):e5527. doi: 10.1371/journal.pone.0005527 (PMC2677457; doi:10.1371/journal.pone.0005527)

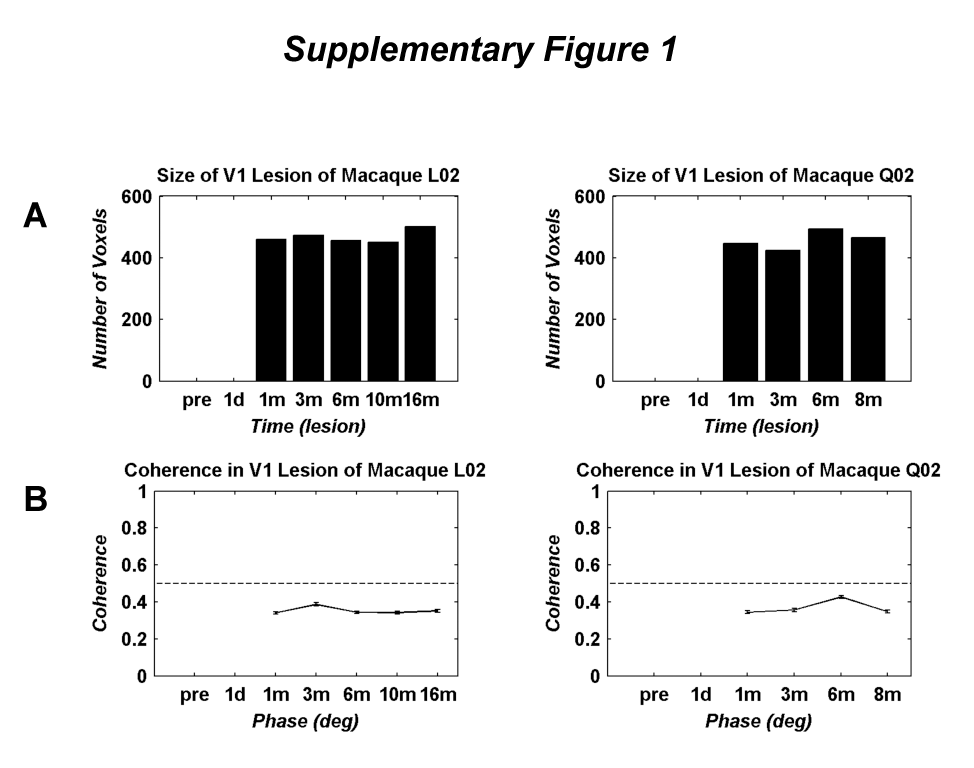

Supplement: Figure S1 — Stability of the V1 lesion. The V1 lesion could be easily identified in each experiment from the anatomical (MDEFT) MR images by the absence of gray matter. A..The number of voxels defining the lesion was plotted over time for monkeys L02 (left) and Q02 (right). B. The mean coherence over all voxels within the lesion was plotted over time for monkeys L02 (left) and Q02 (right). The dashed line corresponds to a coherence level of 1 std>mean noise levels (see methods). The lesion remained stable and inactive over the entire time period. (0.14 MB TIF) [file pone.0005527.s001.tif]

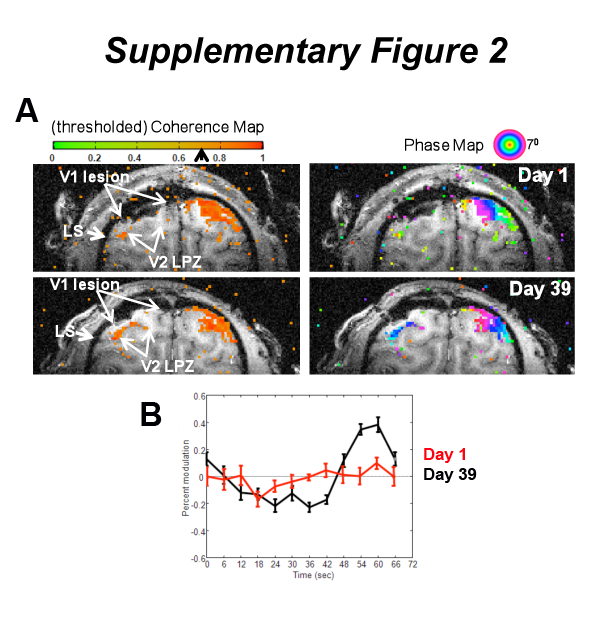

Supplement: Figure S2 — A Comparison of fMRI activity hours versus 39 days post-lesioning. A representative axial slice from the macaque visual cortex with overlaid coherence (left column) and phase (right column) activation maps. The top row represents data obtained on day 1 (∼12 hours after the V1 aspiration lesion), the second row data obtained on day 39. In this slice, the lesion spares a small portion of V1 near the lunate sulcus (which explains the small focus of activity seen outside the foveal border of the V2 LPZ in the upper panels) and extends medially nearly to the midline. Note that on day #1 the V2 LPZ is not significantly modulated by the visual stimulus, in contrast to the strong area V2 modulation seen in the contralateral (intact hemisphere). By day 39, it is clear that weak but significant modulation has returned to the V2 LPZ. B Mean percent signal modulation inside the V2 LPZ during presentation of a ring stimulus centered at 4°. Signal modulation, absent at the first day post-lesioning (red line), recovers within 39 days post-lesioning (black line). (0.26 MB TIF) [file pone.0005527.s002.tif]
